# Longitudinal Genomic Analysis to Fine-tune Targeted Therapy: Results of the Phase II LOGIC 2 Trial in Patients with *BRAF*^V600^-Mutant Metastatic Melanoma

**DOI:** 10.1158/1078-0432.CCR-24-0254

**Published:** 2025-03-19

**Authors:** Reinhard Dummer, Shahneen Sandhu, Wilson H. Miller, Marcus O. Butler, Matthew H. Taylor, Lucie Heinzerling, Christian U. Blank, Eva Muñoz-Couselo, Howard A. Burris, Michael A. Postow, Bartosz Chmielowski, Mark R. Middleton, Carola Berking, Jessica C. Hassel, Anja Heike Gesierich, Cornelia Mauch, Joseph F. Kleha, Anna Polli, Allison S. Harney, Alessandra di Pietro, Paolo A. Ascierto

**Affiliations:** 1 Department of Dermatology, Skin Cancer Unit, University Hospital Zurich, Zurich, Switzerland.; 2 Sir Peter MacCallum Cancer Department of Oncology, University of Melbourne, Melbourne, Australia.; 3 Lady Davis Institute and Segal Cancer Centre, Jewish General Hospital, Montreal, Canada.; 4 Department of Medicine, McGill University, Montreal, Canada.; 5 Department of Oncology, McGill University, Montreal, Canada.; 6 Princess Margaret Cancer Centre, University Health Network, Toronto, Canada.; 7 Department of Medicine, University of Toronto, Toronto, Canada.; 8 Department of Immunology, University of Toronto, Toronto, Canada.; 9 Earle A. Chiles Research Institute, Providence Cancer Institute, Portland, Oregon.; 10 Department of Dermatology and Allergy, University Hospital, Ludwig Maximilian University, Munich, Germany.; 11 Department of Medical Oncology, Netherlands Cancer Institute, Amsterdam, the Netherlands.; 12 Department of Medical Oncology, Melanoma and Other Skin Cancers Unit, Vall d’Hebron Hospital and Vall d’Hebron Institute of Oncology (VHIO), Barcelona, Spain.; 13 Sarah Cannon Research Institute, Nashville, Tennessee.; 14 Department of Medicine, Memorial Sloan Kettering Cancer Center and Weill Cornell Medical College, New York, New York.; 15 Division of Hematology-Oncology, Department of Medicine, Jonsson Comprehensive Cancer Center, University of California Los Angeles, Los Angeles, California.; 16 NIHR Oxford Biomedical Research Centre, Oxford University Hospitals NHS Foundation Trust, John Radcliffe Hospital, Oxford, United Kingdom.; 17 Department of Oncology, University of Oxford, Oxford, United Kingdom.; 18 Early Phase Clinical Trials Unit, Cancer & Haematology Centre, Churchill Hospital, Oxford, United Kingdom.; 19 Department of Dermatology, Uniklinikum Erlangen, CCC Erlangen – EMN, Friedrich-Alexander-University Erlangen-Nürnberg (FAU), Erlangen, Germany.; 20 Department of Dermatology and National Center for Tumor Diseases (NCT), Heidelberg University, Medical Faculty Heidelberg, NCT Heidelberg, A Partnership Between DKFZ and University Hospital Heidelberg, Heidelberg, Germany.; 21 Department of Dermatology, Venerology and Allergology, University Hospital Würzburg, Würzburg, Germany.; 22 Department of Dermatology and Venereology, Faculty of Medicine and University Hospital of Cologne, Cologne, Germany.; 23 Pfizer, New York, New York.; 24 Pfizer, Milan, Italy.; 25 Pfizer Boulder Research Unit, Boulder, Colorado.; 26 Melanoma, Cancer Immunotherapy and Innovative Therapies Unit, Istituto Nazionale Tumori IRCCS Fondazione Pascale, Napoli, Italy.

## Abstract

**Purpose:**

LOGIC 2 (NCT02159066), a multicenter, open-label, two-part, phase II study, assessed encorafenib plus binimetinib combined with a third targeted agent after tumor progression on encorafenib plus binimetinib in patients with locally advanced, unresectable or metastatic *BRAF*^V600^-mutant melanoma.

**Patients and Methods:**

Adults with locally advanced, unresectable or metastatic *BRAF*^V600^-mutant melanoma who were BRAF inhibitor/MEK inhibitor (BRAFi/MEKi) treatment–naïve or pretreated received encorafenib plus binimetinib (part I/run-in). Based on the genomic testing at disease progression following encorafenib plus binimetinib, patients were assigned to one of four treatment arms to receive encorafenib plus binimetinib with an appropriate molecularly targeted agent (ribociclib, infigratinib, capmatinib, or buparlisib; part II). The primary endpoint was best overall response; safety, biomarkers, pharmacokinetics, and other efficacy endpoints were also assessed.

**Results:**

In part I/run-in, 75 BRAFi/MEKi-naïve patients and 83 BRAFi/MEKi-pretreated patients were treated; in part II, 58 patients were treated (ribociclib, *n* = 38; infigratinib, *n* = 1; capmatinib, *n* = 13; buparlisib, *n* = 6). The overall confirmed response rate was 73.3% [95% confidence interval (CI), 61.9–82.9] in BRAFi/MEKi-naïve patients, 25.3% (95% CI, 16.4–36.0) in pretreated patients, 2.6% (95% CI, 0.1–13.8) in the ribociclib arm, and 0% in the other three arms. Adverse events were manageable and consistent with the known safety profile of each drug.

**Conclusions:**

LOGIC 2 supports the use of encorafenib plus binimetinib for treatment-naïve and previously treated, locally advanced, unresectable or metastatic *BRAF*^V600^-mutant melanoma. However, adding a third targeted agent following disease progression did not show meaningful efficacy; further research is needed to identify other therapeutic targets to circumvent resistance.

**Translational Relevance**

The LOGIC 2 (NCT02159066) clinical trial assessed encorafenib plus binimetinib combined with a third targeted agent following tumor progression on encorafenib plus binimetinib treatment in patients with locally advanced, unresectable or metastatic *BRAF*^V600^-mutant melanoma. The clinical efficacy of combining BRAF inhibitor/MEK inhibitor (BRAFi/MEKi) treatment in this population is limited by the development of resistance to treatment. Previous reports indicate possible clinical benefit from re-treatment in some patients with secondary resistance to BRAFi/MEKi combinations. The LOGIC 2 results confirm that patients who were BRAFi/MEKi naïve had a higher response rate than patients who were BRAFi/MEKi pretreated; some patients in the BRAFi/MEKi-pretreated group showed benefit from treatment with encorafenib plus binimetinib. Characterizing genomic alterations following progression may allow for the identification of potential therapeutic targets; however, in this trial, this did not translate into clinical benefit. Further research is needed to identify patterns of resistance susceptible to the addition of novel therapies.

## Introduction

Patients with *BRAF*^V600^-mutated melanoma, the majority of which harbor V600E and V600K mutations, account for approximately 50% of melanoma cases (1, 2). Activating *BRAF* mutations drive MAPK pathway signaling (RAF–MEK1/MEK2–ERK1/ERK2), resulting in melanoma development and progression (3). Combination BRAF inhibitor and MEK inhibitor (BRAFi/MEKi) therapy is an established standard of care for patients with *BRAF*^V600^-mutant metastatic melanoma, including encorafenib plus binimetinib, vemurafenib plus cobimetinib, or dabrafenib plus trametinib (3–8). These combinations are now a well-established standard of care, with improved progression-free survival (PFS) and overall survival (OS) compared with BRAFi monotherapy, with manageable tolerability (5–9).

The ATP-competitive BRAFi encorafenib exhibits unique pharmacology and a dose-dependent effect in melanoma treatment (10, 11). Binimetinib is a potent, selective, allosteric, ATP-uncompetitive inhibitor of MEK1/2 (4, 12, 13). A phase I study of encorafenib determined that the recommended phase II dose was 300 mg once daily (14). A phase Ib/II study demonstrated that encorafenib 450 mg once daily was well tolerated, when combined with binimetinib 45 mg twice daily, which is the recommended phase II dose for subsequent development (10, 12). The combination of encorafenib plus binimetinib has been approved in various countries based on the demonstrated antitumor efficacy in a phase III study (COLUMBUS) of patients with *BRAF*^V600^-mutant metastatic melanoma; PFS [HR, 0.51; 95% confidence interval (CI), 0.40–0.67; median PFS, 14.9 vs. 7.3 months] and OS (HR, 0.64; 95% CI, 0.50–0.81; median OS, 33.6 vs. 16.9 months) were compared with those of the vemurafenib (5, 15).

Despite the high response rate, the clinical efficacy of BRAFi and MEKi combination treatment is limited by the emergence of resistance in almost all patients (16). Characterizing the acquired genomic alterations during BRAFi and MEKi therapy may allow an improved understanding of resistance mechanisms and potentially lead to the identification of new potential therapeutic targets to circumvent resistance. Investigation into the mechanism(s) of resistance is largely limited by the number of genes in each genetic testing panel; various approaches have been taken, including a melanoma-specific multiplex mutational profiling assay developed to detect 43 recurrent mutations occurring in six genes frequently mutated in melanomas (*BRAF*, *NRAS*, *KIT*, *GNAQ*, *GNA11*, and *CTNNB1*; ref. 17). Whole-exome sequencing data have been obtained from 121 melanoma samples (vs. wild-type pairs), with the aim of identifying new mutations that drive melanoma (18). Using this method, 11 genes harboring significant functional mutations were discovered; six (*BRAF*, *NRAS*, *TP53*, *PTEN*, *CDKN2A*, and *MAP2K1*) were already known melanoma-associated genes, and five genes (*PPP6C*, *RAC1*, *SNX31*, *TACC1*, and *STK19*) were further identified. However, cancer cells are continuously acquiring new mutations due to genomic instability and selective pressure from the tissue microenvironment (19–21).

Based on improved accuracy, sensitivity, and high-throughput nature, next-generation sequencing (NGS) has significantly advanced the discovery of cancer genome and transcription characteristics (22). NGS can also be used to determine the allelic fraction of ctDNA and define alterations that recapitulate multisite sampling; studies have shown that ctDNA can be used at baseline as a predictive biomarker of response to targeted therapy (23–26). It can also be an on-treatment biomarker of response and disease progression (PD), as well as a tool to identify mechanisms of resistance (27). Identifying the mechanisms of resistance to BRAFi/MEKi treatment by following the evolution of genomic changes in the tumor environment and studying changes in ctDNA at the time of PD may allow subsequent rational combination therapy to be rapidly initiated (28).

The aim of the current study was to evaluate if NGS-determined genomic profiling of resistant tumors may help with the selection of an additional drug at PD to BRAFi/MEKi that can help overcome resistance in patients with locally advanced or metastatic *BRAF*^V600^-mutant melanoma. An exploratory objective was to assess changes in *BRAF*^V600^ concentrations in ctDNA using NGS at PD.

## Patients and Methods

### Study design and participants

LOGIC 2 (NCT02159066), a multicenter, open-label, two-part, phase II study, assessed the efficacy and safety of encorafenib plus binimetinib (part I) followed by encorafenib plus binimetinib combined with a third targeted agent after tumor progression on encorafenib plus binimetinib (part II) in patients with locally advanced, unresectable or metastatic *BRAF*^V600^-mutant melanoma.

**Figure 1.**
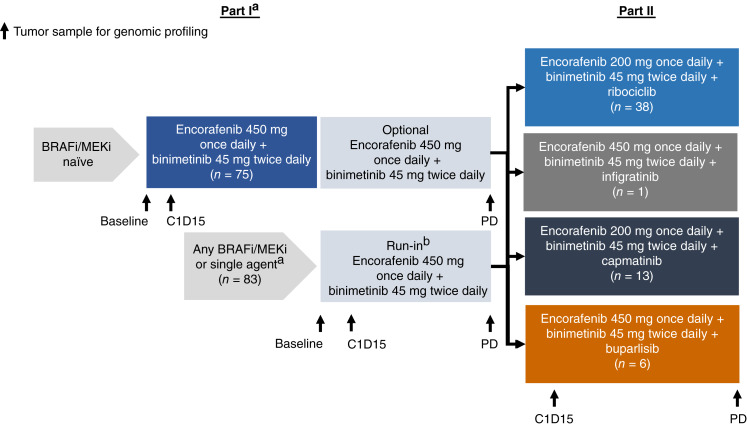
LOGIC 2 study design. A multicenter, open-label, two-part, phase II study to assess the efficacy and safety of encorafenib plus binimetinib (part I) followed by encorafenib plus binimetinib combined with a third targeted agent after tumor progression (part II) in patients with locally advanced, unresectable or metastatic *BRAF*^V600^-mutant melanoma. ^a^Single agents include vemurafenib, dabrafenib, encorafenib, trametinib, and binimetinib; combinations include dabrafenib/trametinib and encorafenib/binimetinib. ^b^Patients who experienced PD on a prior BRAFi/MEKi regimen continued encorafenib/binimetinib if a PR was observed followed by a new biopsy at progression. Patients who did not progress on their prior regimen could continue the encorafenib/binimetinib combination until evidence of PD, at which point a tumor biopsy would be taken and analyzed to guide assignment to a triplet combination arm in part II.

In part I/run-in, two groups of patients were treated with encorafenib 450 mg once daily plus binimetinib 45 mg twice daily until PD. Group A consisted of patients who were naïve for selective BRAFis and MEKis; Group B comprised patients who had prior exposure to selective BRAFis and/or MEKis, except for patients who had PD after prior encorafenib plus binimetinib treatment (Fig. 1). Patients from both groups in part I/run-in were enrolled into part II at the time of documented PD so long as they met the inclusion criteria for part II.

Eligible patients, aged ≥18 years, had a histologically confirmed diagnosis of unresectable stage III or metastatic melanoma stage IIIC to IV per the American Joint Committee on Cancer guidelines (29), had documented evidence of a *BRAF*^V600^ mutation, had evidence of measurable disease, per RECIST version 1.1 (30), and Eastern Cooperative Oncology Group (ECOG) performance status (PS) of ≤2 (31). In part I (group A only), patients provided an archival or newly obtained tumor sample at baseline and agreed to a mandatory biopsy at the time of progression from the encorafenib plus binimetinib combination, if not medically contraindicated. Patients who had not received prior selective BRAFi and MEKi treatment (naïve) and may have received other permitted treatments, such as chemotherapy (e.g., anthracyclines and taxanes) or biological therapy (e.g., mAbs and protein kinase inhibitors), were eligible. In part I/run-in (group B only), patients with PD after treatment with a single-agent BRAFi or MEKi or the combination of BRAFi/MEKi (excluding the encorafenib plus binimetinib combination) and patients who had not progressed on a prior BRAFi and/or MEKi regimen (including encorafenib and/or binimetinib), but did not tolerate this treatment, were able to enter group B upon consultation with the sponsor.

Tumor biopsies at baseline and on PD were sequenced using technologies such as NGS. Based on the changes in genomic alterations observed in tumor tissue biopsies obtained at baseline and at PD, patients were assigned to one of four treatment arms in part II of the study. Patients continued to receive encorafenib plus binimetinib in addition to a third individually selected molecularly targeted agent (triplet combinations); ribociclib [cyclin-dependent kinase (CDK) 4 and 6 inhibitor] was given at the previously defined MTD, whereas dose escalations were used in the infigratinib (FGFR inhibitor), capmatinib (MET-targeting kinase inhibitor), and buparlisib (PI3K inhibitor) arms. Patients with *CCND1* amplification, *CDK4* amplification or mutation, *B/CRAF* amplification, *MAP2K1/K2* mutation, *N/K/HRAS* mutation, *P16* loss, or no alterations in any genes mentioned for any of the following arms received encorafenib (200 mg once daily) plus binimetinib (45 mg twice daily) and ribociclib (600 mg once daily, given 3 weeks on/1 week off). Patients with *HER2* amplification, *IGF-1R* amplification, *EGFR* amplification or mutation, *PIK3CA* mutation, or *PTEN* mutation or loss received encorafenib (450 mg once daily) plus binimetinib (45 mg twice daily) and buparlisib (60 or 90 mg, once daily). Patients with *cMET* amplification received encorafenib (200 mg once daily) plus binimetinib (45 mg twice daily) and capmatinib (200, 300, 400, or 600 mg once daily). Patients with *FGFR1/2/3* amplifications or mutations received encorafenib (450 mg once daily) plus binimetinib (45 mg twice daily) and infigratinib (75 mg, once daily). If no relevant alteration was identified, the investigator, in discussion with the medical monitor and biomarker lead, assigned patients to the group deemed most appropriate for the patient.

The final protocol, amendments, and informed consent documentation were reviewed and approved by institutional review boards and independent ethics committees at each participating center. Investigators were required to inform their institutional review boards or independent ethics committees of the study’s progress and the occurrence of any serious or unexpected adverse events (AE). This study was conducted in compliance with the Declaration of Helsinki, the International Conference on Harmonization Guideline for Good Clinical Practice, and applicable local regulatory requirements. Informed written consent was obtained from each participant or each participant’s guardian.

### Study endpoints

The primary efficacy endpoint was overall response as measured by the overall response rate (ORR) and as determined by investigator-assessed tumor evaluations per RECIST version 1.1. The secondary efficacy endpoints included PFS, duration of response (DOR), disease control rate (DCR), time to response (TTR), and OS (part II only). The safety and tolerability of encorafenib and binimetinib were assessed by the incidence and severity of AEs and serious AEs (SAE) according to Common Terminology Criteria for Adverse Events version 4.03 (32). The incidence of dose-limiting toxicities in cycle 1 of the triplet combinations was also assessed in part II.

### Biomarker analysis

Tumor samples were collected prior to treatment with the encorafenib plus binimetinib combination, then at cycle 1, day 15 and PD in part I/run-in and part II; archival samples were allowed, if available. Genomic profiling in tumor tissue was performed using an NGS panel of 324 genes [e.g., mutation, amplification, deletion, and rearrangements (FoundationOneCDx; Foundation Medicine Inc.)] at baseline and progression (part I) for potential predictive markers and acquired resistance. A droplet digital PCR BEAMing assay was performed at Sysmex Inostics (RRID: SCR_025629). Changes in *BRAF*^V600E^ concentrations were assessed in ctDNA from patients at baseline, on treatment, and at PD during the encorafenib plus binimetinib combination and triplet combination treatments in parts I and II; the assay only captured *BRAF*^V600E/K^ mutations. Prior to this study, treatment with encorafenib and binimetinib plus ribociclib in a specific subgroup of patients depending on their genetic mutation had not been investigated. Therefore, it was decided that investigators could select additional participants with mutations outside of those defined in the study protocol (including *CDK4*, *MAP2K1/K2*, *N/KRAS*, and *HRAS*) for the ribociclib arm, based on whether the investigator believed participants could experience clinical benefit from this treatment.

### Pharmacokinetics

During part I/run-in of the study, pharmacokinetic (PK) blood samples from patients treated with encorafenib and binimetinib were collected, and PK sampling scheduled on cycle 1, days 1 (after dose) and 15 (prior to and after dose); cycle 2, days 8 and 21 (prior to dose), then day 15 of subsequent cycles up to cycle 5 (prior to dose); and at the end of study treatment. During part II of the study, the PK of encorafenib, binimetinib (including its primary active metabolite AR00426032), capmatinib, and buparlisib were assessed; sampling schedules included cycle 1, days 1, 8, 15, and 16; cycle 2, days 1 and 15, then day 1 of subsequent cycles up to cycle 5; and at the end of study treatment. The PK of encorafenib, binimetinib (including its primary active metabolite AR00426032), ribociclib (including its active metabolite LEQ803), and infigratinib (including its active metabolites BHS697 and CQM157) were assessed, and the sampling schedules included cycle 1, days 1, 8, 15, 16, and 21; cycle 2, days 1 and 15, then day 1 of subsequent cycles up to cycle 5; and at the end of study treatment.

PK samples were collected and evaluated for all patients enrolled in both parts of the study for the first five cycles or until they withdrew consent. Plasma concentrations of study drugs and their metabolites (as applicable) were measured by a designated contract research organization (WuXi AppTec Co., Ltd., RRID: SCR_001217) using a validated LC/MS-MS assay. The lower limit of quantitation was 1.0 ng/mL for encorafenib, binimetinib, capmatinib, and infigratinib; 0.25 ng/mL for buparlisib; and 5.0 ng/mL for ribociclib.

### Statistical analyses

The full analysis set (FAS) included all patients who received at least one dose (partial or full) of encorafenib or binimetinib (part I) and at least one dose of encorafenib, binimetinib, or the third agent (part II) and was used for the analysis of all endpoints unless otherwise noted. The safety set included all patients in the FAS who had at least one valid postbaseline safety assessment. The per protocol set included patients in part II in the FAS who were compliant with the protocol.

The best overall response (BOR) was the best response recorded from the start of the treatment until PD or clinical progression, death, or early study discontinuation, whichever occurred first. The BOR was used to evaluate the tumor response in terms of the overall response, based on investigator-assessed tumor evaluations per RECIST version 1.1 (30). ORR was defined as the proportion of participants with a confirmed BOR of complete response (CR) or partial response (PR). DCR was defined as the proportion of participants with a BOR of CR, PR, or stable disease. ORR and DCR were provided for all patients with a corresponding 95% CI based on the Clopper–Pearson method.

For PFS, patients who had not progressed or had died at the time of the final data cutoff were censored at the date of their last adequate tumor assessment, unless this was unknown. PFS was defined as the start date of the study drug (or study treatment for part II) until first documented PD or death due to any cause. A Kaplan–Meier plot for PFS was produced. The median PFS (in months), 25th and 75th percentiles with corresponding 95% CIs, and Kaplan–Meier–estimated probabilities with corresponding 95% CIs at several timepoints (including 6, 12, 18, and 24 months) were included (33).

OS was defined as the time from the start date of treatment to the date of death from any cause. If a patient was not known to have died, survival was censored at the last known date the patient was alive. TTR was calculated for confirmed responders only and was defined as the time between the start of study treatment and the first documented response (CR or PR) that was subsequently confirmed. DOR was calculated for confirmed responders only and was defined as the time between the date of the first documented response (CR or PR) and the date of first documented progression or death due to underlying cancer. Kaplan–Meier plots with median and 95% CIs were reported for OS, TTR, and DOR.

The safety summary tables (except deaths) only included assessments collected no later than 30 days after study treatment discontinuation. All safety assessments were listed, and those collected later than 30 days after study treatment discontinuation were highlighted.

Data were summarized with respect to demographic and baseline characteristics, efficacy observations and measurements, safety observations and measurements, biomarker measurements, and PK measurements. Data from participating centers were combined and analyzed using SAS version 9.2 or higher (SAS Institute Inc., RRID: SCR_008567).

### Data availability

Upon request, and subject to review, Pfizer will provide the data that support the findings of this study. Subject to certain criteria, conditions, and exceptions, Pfizer may also provide access to the related individual de-identified participant data. See https://www.pfizer.com/science/clinical-trials/trial-data-and-results for more information.

## Results

One hundred and fifty-eight patients were enrolled (first patient first visit: July 23, 2014; last patient first visit: March 14, 2018; last patient last visit: January 13, 2023) and treated with encorafenib plus binimetinib in part I/run-in of the study; 75 patients had not previously received BRAFi/MEKi (naïve), and 83 patients had received BRAFi/MEKi (other than encorafenib plus binimetinib) previously (pretreated). Fifty-eight patients were enrolled in part II and received study treatment; 38 patients were treated with encorafenib plus binimetinib and ribociclib, 1 with encorafenib plus binimetinib and infigratinib, 13 with encorafenib plus binimetinib and capmatinib, and 6 with encorafenib plus binimetinib and buparlisib (Table 1). Prior phase I studies comparing the triplet combinations of encorafenib plus binimetinib plus ribociclib, infigratinib, buparlisib, or capmatinib have not been completed in this setting. The buparlisib arm was closed during the study because of drug–drug interactions with encorafenib. The one patient in the infigratinib triplet died because of PD, corresponding to an OS of 20.8 months.

**Table 1.** Patient disposition (FAS population, part I/run-in, and part II).

	Part I/run-in		Part II		
Patient disposition, *n* (%)	Encorafenib + binimetinib (naïve)	Encorafenib + binimetinib (pretreated)	Encorafenib + binimetinib + ribociclib	Encorafenib + binimetinib + capmatinib	Encorafenib + binimetinib + buparlisib
	*n* = 75	*n* = 83	*n* = 38	*n* = 13	*n* = 6
Treatment ongoing	0	0	0	0	0
Treatment discontinued	75 (100.0)	83 (100.0)	38 (100.0)	13 (100.0)	6 (100.0)
Primary reason for treatment discontinuation					
AE	9 (12.0)	5 (6.0)	1 (2.6)	1 (7.7)	1 (16.7)
Progressive disease	42 (56.0)	47 (56.6)	35 (92.1)	8 (61.5)	4 (66.7)
Death	7 (9.3)	9 (10.8)	1 (2.6)	2 (15.4)	1 (16.7)
Physician decision	5 (6.7)	10 (12.0)	1 (2.6)	0	0
Patient/guardian decision	9 (12.0)	4 (4.8)	0	2 (15.4)	0
Study terminated by the sponsor	3 (4.0)	3 (3.6)	0	0	0

Baseline patient and disease characteristics for part I/run-in and part II are shown in Supplementary Tables S1 and S2, and the representativeness of the study population is described in Supplementary Table S3. In part I/run-in, the baseline characteristics were generally balanced between the naïve and pretreated groups in the FAS population. Both groups had a higher proportion of males (62.7% and 53.0%, respectively). The median age in the naïve and pretreated groups was 56 years (range, 23–80) and 53 years (range, 29–83), respectively. The two groups differed when it came to ECOG PS: in part I/run-in, most of the patients in the naïve group had an ECOG PS 0 (73.3%), whereas 49.4% had an ECOG PS 0 in the pretreated group. In both groups, most patients had stage IV disease (80.0% and 85.5%, respectively) at the time of study entry.

Forty percent of naïve patients in part I/run-in had received prior antineoplastic therapy; the most frequently received therapies were ipilimumab (21.3%) and IFN (10.7%). The most frequent BRAFi received by pretreated patients in part I/run-in was dabrafenib (51.8%), whereas trametinib was the most frequent MEKi (36.1%); the most frequent prior anticancer therapy, apart from BRAFi/MEKi, was ipilimumab (51.8%; Supplementary Table S4).

### Efficacy

In part I/run-in, the ORR was 73.3% (95% CI, 61.9–82.9) in BRAFi/MEKi-naïve patients and 25.3% (95% CI, 16.4–36.0) in pretreated patients; the respective DCRs were 92.0% (95% CI, 83.4–97.0) and 42.2% (95% CI, 31.4–53.5; Table 2), respectively. In part II, the ORR was 2.6% (95% CI, 0.1–13.8) for the encorafenib plus binimetinib and ribociclib triplet, and 0% for the other triplet combinations. The DCRs were 26.3% (95% CI, 13.4–43.1), 0% (95% CI, not available), 15.4% (95% CI, 1.9–45.4), and 16.7% (95% CI, 0.4–64.1) for the ribociclib, infigratinib (data not shown), capmatinib, and buparlisib triplet combinations, respectively (Table 2). In the encorafenib plus binimetinib and ribociclib triplet per protocol set, the ORR and DCR were 2.8% (95% CI, 0.1–14.5) and 27.8% (95% CI, 14.2–45.2), respectively. Supplementary Figure S1 shows the best percentage change from baseline in target lesions as per local assessment.

**Table 2.** BOR as per local assessment (FAS population, part I/run-in, and part II).

	Part I/run-in		Part II		
Response type	Encorafenib + binimetinib (naïve)	Encorafenib + binimetinib (pretreated)	Encorafenib + binimetinib + ribociclib	Encorafenib + binimetinib + capmatinib	Encorafenib + binimetinib + buparlisib
	*n* = 75	*n* = 83	*n* = 38	*n* = 13	*n* = 6
BOR, *n* (%)^a^,^b^					
CR	10 (13.3)	3 (3.6)	0	0	0
PR	45 (60.0)	18 (21.7)	1 (2.6)	0	0
SD	14 (18.7)	14 (16.9)	9 (23.7)	2 (15.4)	1 (16.7)
PD	4 (5.3)	30 (36.1)	26 (68.4)	7 (53.8)	3 (50.0)
Unknown	2 (2.7)	16 (19.3)	1 (2.6)	4 (30.8)	2 (33.3)
Not assessed	0	2 (2.4)	1 (2.6)	0	0
ORR (95% CI), %^c^	73.3 (61.9–82.9)	25.3 (16.4–36.0)	2.6 (0.1–13.8)	0 (0.0–24.7)	0 (0.0–45.9)
DCR (95% CI), %^c^	92.0 (83.4–97.0)	42.2 (31.4–53.5)	26.3 (13.4–43.1)	15.4 (1.9–45.4)	16.7 (0.4–64.1)

Abbreviation: SD, stable disease.

a BOR was based on local assessment using RECIST version 1.1.

b CR and PR were confirmed by repeated assessments performed not less than 4 weeks after the criteria for response were first met.

c The 95% CI for the frequency distribution of each variable was computed using the Clopper–Pearson method.

**Figure 2.**
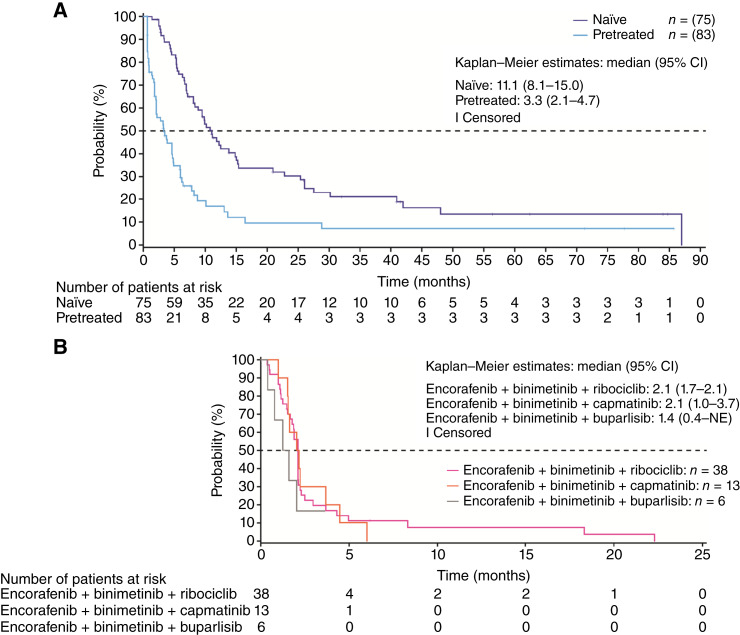
PFS in (**A**) part I/run-in and (**B**) part II. **A,** Kaplan–Meier analysis of PFS with encorafenib plus binimetinib in naïve and pretreated patients in the FAS population. **B,** Kaplan–Meier analysis of PFS in the encorafenib plus binimetinib and ribociclib, encorafenib plus binimetinib and capmatinib, and encorafenib plus binimetinib and buparlisib arms.

In part I/run-in, the median PFS was 11.1 months (95% CI, 8.1–15.0) in naïve patients and 3.3 months (95% CI, 2.1–4.7) in pretreated patients (Fig. 2A). In part II, the median PFS was 2.1 months (95% CI, 1.7–2.1), 2.1 months (95% CI, 1.0–3.7), and 1.4 months [95% CI, 0.4–not evaluable (NE)] for the ribociclib, capmatinib, and buparlisib triplet combinations, respectively (Fig. 2B); the PFS for the one patient in the encorafenib plus binimetinib and infigratinib arm was 2.1 months (data not shown). In the ribociclib triplet per protocol set, the PFS was 2.1 months (95% CI, 1.8–2.1).

In part I/run-in, the median DOR was 10.9 months (95% CI, 8.1–14.1; *n* = 55) for naïve patients and 5.6 months (95% CI, 3.9–13.0; *n* = 21) for pretreated patients. In part II, one patient in the ribociclib triplet achieved a BOR of PR; the DOR was 2.1 months, and the TTR was 4.1 months (Supplementary Fig. S2).

In part II, the median OS was 10.4 months (95% CI, 6.0–16.7), 5.6 months (95% CI, 1.7–NE), and 2.5 months (95% CI, 0.4–NE) for the ribociclib, capmatinib, and buparlisib triplet combinations.

### Biomarker

In patients in part I/run-in, *BRAF*^V600^ variants were identified in baseline tumor tissue from 112 of 158 (70.9%) patients, with V600E being most common (99/112; 88.4%), followed by V600K (11/112; 9.8%), and V600G and V600R being present in only one patient each (1/112; 0.9%). The other genomic alterations found in more than 5% of the patients at baseline were *BRAF* amplification (13/158; 8.2%), *MET* amplification (10/158; 6.3%), and *PTEN* loss (17/158; 10.8%). Similar molecular alterations were also observed in end-of-treatment tumor tissue samples (Supplementary Table S5).

ctDNA analysis data were obtained from 75 BRAFi/MEKi-naïve patients; 39 patients had *BRAF*^V600E^ followed by no mutation detected (NMD), and 10 patients had no change in detectable *BRAF*^V600E^ mutation (*BRAF*^V600E^–*BRAF*^V600E^). The response rate was 73.3% (95% CI, 61.9–82.9) for all naïve patients with ctDNA results, 82.1% (95% CI, 66.5–92.5) for patients with *BRAF*^V600E^–NMD, and 60.0% (95% CI, 26.2–87.8) for patients with *BRAF*^V600E^–*BRAF*^V600E^ mutations (Table 3).

**Table 3.** Change in ctDNA *BRAF*^V600E^: BRAFi/MEKi naïve vs. pretreated.

	Total participants*, n*	Overall response, *n* (%)	95% CI^a^
BRAFi/MEKi naïve			
All BRAFi/MEKi naïve	75	55 (73.3)	61.9–82.9
*BRAF*^V600E^–NMD	39	32 (82.1)	66.5–92.5
*BRAF*^V600E^–*BRAF*^V600E^	10	6 (60.0)	26.2–87.8
Prior BRAFi or MEKi therapy			
All prior BRAFi or MEKi	78	20 (25.6)	16.4–36.8
*BRAF*^V600E^–NMD	12	8 (66.7)	34.9–90.1
*BRAF*^V600E^–*BRAF*^V600E^	27	4 (14.8)	4.2–33.7

*BRAF* ^V600E^–NMD indicates that *BRAF*^V600E^ ctDNA was detected at baseline and not at cycle 1 day 15.

a The 95% CI for the frequency distribution of each variable was computed using the Clopper–Pearson method.

ctDNA analysis data were obtained from 78 BRAFi/MEKi-pretreated patients; 12 patients had *BRAF*^V600E^–NMD, and 27 patients had *BRAF*^V600E^–*BRAF*^V600E^ mutations. The response rate was 25.6% (95% CI, 16.4–36.8) for all pretreated patients with ctDNA results, 66.7% (95% CI, 34.9–90.1) for patients with *BRAF*^V600E^–NMD, and 14.8% (95% CI, 4.2–33.7) for patients with *BRAF*^V600E^–*BRAF*^V600E^ mutations (Table 3).

In part II, 29 genetic alterations were identified at enrollment (Supplementary Table S6). In the encorafenib plus binimetinib and ribociclib arm, these were *KRAS* (A146V, *n* = 1), *NRAS* (Q61R, *n* = 2; Q61K, *n* = 4), *HRAS* (G13R, *n* = 1), *CDKN2A* (splice site 151-1G>A, *n* = 2; V126D, *n* = 1; D146fs*12+, *n* = 1; E61*, *n* = 1; Y44fs*1, *n* = 1; and loss of copy number, *n* = 7), *BRAF* (amplification, *n* = 2), *CDK4* (R24H, *n* = 1), and *MAP2K1* (F531, *n* = 1). In the encorafenib plus binimetinib and capmatinib arm, these were *MET* amplifications (*n* = 2). In the encorafenib plus binimetinib and buparlisib arm, these were *PTEN* loss of copy number (*n* = 1) and *PIK3CA* (M1043V, *n* = 1). There were 51 new alterations identified at progression in the triplet combinations (Supplementary Table S7). These were *BRAF* (V600E, *n* = 3; V600K, *n* = 1; rearrangement, *n* = 5; and amplification, *n* = 4), *CDKN2A* (L64_H66del, *n* = 1; loss of copy number, *n* = 7), *H/N/KRAS* (*KRAS*^A146V^, *n* = 1; *NRAS*^Q61R^, *n* = 2; *NRAS*^Q61K^, *n* = 4; and *HRAS*^G13R^, *n* = 1), *TERT* promoter (-146C>T, *n* = 5; *-*124C>T, *n* = 1), *CDKN2B* loss of copy number (*n* = 5), *PTEN* amplification (*n* = 4), *CDK6* amplification (*n* = 3), *HGF* amplification (*n* = 3), and *KEL* amplification (*n* = 3).

### Safety

The median duration of exposure in part I/run-in was 47.6 weeks (range, 3.9–403.7) for the naïve group and 13.0 weeks (range, 0.1–385.3) for the pretreated group (Supplementary Table S8). In part II, the median duration of exposure was 9.7 weeks (range, 0.9–97.0) for the encorafenib plus binimetinib and ribociclib triplet, 9.1 weeks (range, 3.0–38.7) for the encorafenib plus binimetinib and capmatinib triplet, and 5.0 weeks (range, 0.9–14.9) for the encorafenib plus binimetinib and buparlisib triplet (Supplementary Table S9).

A summary of AEs for part I/run-in and part II is reported in Table 4. In part I/run-in, AEs occurred in 157 (99.4%) patients and 135 (85.4%) patients had AEs suspected to be study drug related (Supplementary Table S10). SAEs that occurred regardless of study treatment are summarized in Supplementary Table S11. AEs leading to permanent discontinuation of study treatment occurred in 23 (14.6%) patients (Supplementary Table S12). AEs leading to drug interruption and/or dose adjustment occurred in 102 (64.6%) patients (Supplementary Table S13). AEs requiring additional therapies occurred in 147 (93.0%) patients.

**Table 4.** Overall summary of deaths and AEs (safety set, part I/run-in, and part II).

	Part I/run-in				Part II					
	Encorafenib + binimetinib (naïve)		Encorafenib + binimetinib (pretreated)		Encorafenib + binimetinib + ribociclib		Encorafenib + binimetinib + capmatinib		Encorafenib + binimetinib + buparlisib	
	*n* = 75		*n* = 83		*n* = 38		*n* = 13		*n* = 6	
*n* (%)	All grades	Grade 3/4	All grades	Grade 3/4	All grades	Grade 3/4	All grades	Grade 3/4	All grades	Grade 3/4
All deaths^a^	20 (26.7)		29 (34.9)		28 (73.7)		8 (61.5)		4 (66.7)	
On-treatment deaths^b^	11 (14.7)		13 (15.7)		5 (13.2)		3 (23.1)		4 (66.7)	
AEs	75 (100)	62 (82.7)	82 (98.8)	64 (77.1)	37 (97.4)	25 (65.8)	12 (92.3)	9 (69.2)	6 (100)	6 (100)
Suspected to be drug related	71 (94.7)	30 (40.0)	70 (84.3)	33 (39.8)	32 (84.2)	11 (28.9)	10 (76.9)	7 (53.8)	3 (50.0)	1 (16.7)
SAEs	54 (72.0)	47 (62.7)	53 (63.9)	47 (56.6)	19 (50.0)	15 (39.5)	6 (46.2)	5 (38.5)	4 (66.7)	4 (66.7)
Suspected to be drug related	13 (17.3)	9 (12.0)	15 (18.1)	11 (13.3)	2 (5.3)	2 (5.3)	3 (23.1)	2 (15.4)	0	0
AEs leading to discontinuation	11 (14.7)	6 (8.0)	12 (14.5)	8 (9.6)	4 (10.5)	3 (7.9)	2 (15.4)	2 (15.4)	1 (16.7)	0
Suspected to be drug related	7 (9.3)	2 (2.7)	7 (8.4)	4 (4.8)	2 (5.3)	2 (5.3)	0	0	1 (16.7)	0
AEs requiring dose interruption and/or change	52 (69.3)	39 (52.0)	50 (60.2)	33 (39.8)	13 (34.2)	9 (23.7)	9 (69.2)	4 (30.8)	3 (50.0)	3 (50.0)
Suspected to be drug related	35 (46.7)	18 (24.0)	35 (42.2)	22 (26.5)	10 (26.3)	7 (18.4)	6 (46.2)	3 (23.1)	1 (16.7)	1 (16.7)
AEs requiring additional therapy^c^	71 (94.7)	50 (66.7)	76 (91.6)	50 (60.2)	33 (86.8)	19 (50.0)	10 (76.9)	4 (30.8)	6 (100)	4 (66.7)
Suspected to be drug related	57 (76.0)	14 (18.7)	57 (68.7)	18 (21.7)	14 (36.8)	5 (13.2)	8 (61.5)	3 (23.1)	1 (16.7)	0

One participant who received encorafenib plus binimetinib and infigratinib (not shown in Table 4) experienced one nontreatment-related AE and death.

a All deaths, regardless of cause, including those occurring >30 days after the end of treatment.

b Deaths occurring >30 days after the end of treatment are not included.

c Additional therapy includes all nondrug therapy and concomitant medications.

In part II, AEs occurred in 37 (97.4%), 12 (92.3%), and 6 (100%) patients in the encorafenib plus binimetinib and ribociclib, encorafenib plus binimetinib and capmatinib, and encorafenib plus binimetinib and buparlisib arms, respectively; AEs suspected to be study drug related occurred in 84.2%, 76.9%, and 50.0%, respectively (Supplementary Table S14).Grade 3/4 AEs occurred in 25 (65.8%), 9 (69.2%), and 6 (100%) patients, respectively. SAEs are described in Supplementary Table S15. AEs leading to discontinuation and/or dose adjustment are reported in Supplementary Tables S16 and S17, respectively. AEs requiring additional therapies occurred in 50 patients, including 33 (86.8%) patients in the encorafenib plus binimetinib and ribociclib arm, 1 (100%) in the encorafenib plus binimetinib and infigratinib arm, 10 (76.9%) in the encorafenib plus binimetinib and capmatinib arm, and 6 (100%) in the encorafenib plus binimetinib and buparlisib arm. Dose-limiting toxicities in cycle 1 are reported in Supplementary Table S18.

### Pharmacokinetics

Substantial changes were not observed for mean plasma concentrations of encorafenib, binimetinib, or AR00426032 on day 15 in patients who received encorafenib and binimetinib plus a third agent, following the repeated administration of triplet combinations during cycle 1.

In the encorafenib plus binimetinib and capmatinib arm, encorafenib and binimetinib steady-state plasma exposures were slightly higher when combined with higher doses of capmatinib (300 and 400 mg) compared with lower capmatinib dose combinations (200 mg). Co-administration of a higher dose of capmatinib (300 mg) resulted in a higher maximum observed plasma concentration after drug administration at steady state and area under the concentration–time curve from time zero to time tau at steady state and lower apparent total plasma clearance of drug after oral administration of encorafenib and binimetinib at steady state.

## Discussion

The combination of encorafenib plus binimetinib provided a clinically meaningful antitumor response in patients with locally advanced, unresectable or metastatic *BRAF*^V600^-mutant melanoma. Clinical benefits with this combination therapy included induction of durable tumor responses and a tolerable, manageable safety profile. These data were in keeping with previous encorafenib plus binimetinib data (5).

As expected, the ORR and DCR were lower, and the median PFS was shorter in patients who had received prior BRAFi/MEKi compared with patients who had not. At the first evaluation of response, patients who had received prior BRAFi/MEKi had a lower rate of decrease in *BRAF*^V600E^ ctDNA than patients with no prior BRAFi/MEKi. These results showed that there are some patients who benefit from treatment with encorafenib plus binimetinib after prior exposure to different BRAFi/MEKi combinations, which aligns with the results from previous studies (34–36).

The fundamental premise of LOGIC 2 was that genetic alterations such as mutations, rearrangements, and gain and loss of copy number in previously described pathways implicated in acquired resistance could be identified after progression on BRAFi/MEKi and that this information could be used in real time to inform a rationally selected addition of a third therapeutic agent in part II to circumvent the resistance. Although part II of LOGIC 2 suggested that the addition of a third agent to encorafenib plus binimetinib at PD based on the genetic alterations identified in the progressing tumor sample was feasible with acceptable safety—as the study was able to identify targetable mutations and showed a safety profile consistent with the known profile of encorafenib plus binimetinib—the antitumor activity observed from this approach was very low and not clinically meaningful. Therefore, further exploration to identify pertinent patterns of resistance susceptible to additional therapeutic targeting is warranted. For example, the emergence of small populations of persistent cells selected during targeted therapy may drive resistance. Although some of the underlying mechanisms that define intrinsic resistance to anticancer drugs are likely to be genomic, others may occur at a transcriptomic level and may not be adequately captured on NGS (37). One of the more common nongenomic mechanisms of acquired resistance is the reactivation of the RAF/MEK/ERK pathway in the presence of an inhibitor (38–40). Acquired resistance to BRAFis may arise as a result of secondary signaling changes involving the activation of EGFR, alternative *BRAF* mRNA splicing variants or COT/Tpl2 (encoded by *MAP3K8*), and activation of *CRAF* (41–43). Consistent with these observations, combining selective RAF inhibitors and MEKis was postulated to confer a more durable response than single-agent treatments (6, 44, 45). In this study, the combination of encorafenib plus binimetinib as part of triplet therapy for *BRAF*^V600E^-mutant metastatic melanoma supports this approach.

Participants who were assigned to the ribociclib arm had no clear indication for any of the other treatment arms. As such, the triplet ribociclib arm included patients whose tumors had a nontargetable alteration profile based on the knowledge that p14/p16-based cell-cycle control is an essential driver in melanoma evolution and progression. Interrupting encorafenib, binimetinib, and ribociclib dosing in part II of the study could have disrupted any potential benefit to the participants from this study treatment. Therefore, the frequency of *CDK4* or *RAS* mutations in this treatment arm was not considered at progression, and the antitumor status of the participants treated with the triple combination treatment that included ribociclib was not discussed in this study.

PK analysis highlighted that encorafenib and binimetinib exposures were generally unchanged with the addition of a third agent, with respect to higher maximum observed plasma concentrations after drug administration at steady state and time zero to time tau at steady state after repeated triple administration in cycle 1. However, definitive conclusions could not be drawn because of the limited number of patients included in the PK assessment of some triplet combinations. Additionally, there were limited data for the changes from baseline of pharmacodynamic markers in tumor tissue (e.g., phospho-ERK, phospho-AKT, and DUSP6); therefore, these data are not reported, and no definitive conclusions can be made.

In both parts of the study, AEs were manageable, suggesting that the benefit–risk ratio of treatment in this patient population was favorable, and AEs observed were consistent with the known safety profile of the combination of encorafenib plus binimetinib in patients with a *BRAF* mutation.

The innovative conceptional framework of LOGIC 2 was intended to use NGS to identify putative resistance mechanisms and to target these pathways with the addition of a third agent in real time. However, the study faced limitations during implementation. The approach taken in LOGIC 2 illustrates the importance of considering the challenges of selecting therapies based on genomic testing. Investigation of the mechanism(s) of resistance was limited by the number of genes examined by the genetic assay used, with many resistance mechanisms at the transcriptional, protein, or regulatory level, and therefore not evident using genomic analysis. Additionally, tumor heterogeneity limits the results of genomic testing when trying to assess tumor resistance based on one tumor biopsy (46). At the time the study was designed, it was hoped that this could be overcome; however, the molecular tools did not provide the guidance required for this difficult-to-treat therapy-resistant population. Therefore, the majority of patients in part II were enrolled into the ribociclib triplet arm because of the lack of a targetable mutation. The ctDNA NGS panel used in LOGIC 2 captured *BRAF*^V600E/K^ mutations only, and sequencing for other mutations was not available. Therefore, consistency between ctDNA analysis and tumor tissue analysis was not achievable. ctDNA sequencing has improved in recent years, and it may be possible to map out emerging resistant clones using longitudinal ctDNA sampling and deep whole-genome sequencing in future studies (47).

Another potential limitation of the study design is that it did not include immune checkpoint inhibitors or an immune checkpoint inhibitor–experienced patient population. Currently, immune checkpoint inhibitors demonstrate superior OS in *BRAF*^V600E^-mutant melanoma (48), due in part to the nearly inevitable emergence of resistance to kinase inhibitors. Potential methods for overcoming kinase inhibitor resistance remain a key area of focus within the melanoma landscape (49). Therefore, were this study designed today, patients previously treated with immune checkpoint inhibitors would be included to address the remaining unmet need of overcoming resistance to treatment.

In conclusion, LOGIC 2 supports the use of encorafenib plus binimetinib in patients with naïve and previously treated, locally advanced, unresectable or metastatic *BRAF*^V600^-mutant melanoma and highlighted the feasibility of adding a third agent. However, further research is needed to identify patterns of resistance susceptible to the addition of a third agent.

## Supplementary Material

Supplementary Data 1 Longitudinal Genomic Analysis to Fine-Tune Targeted Therapy: Results of the Phase II LOGIC 2 Trial in Patients With BRAF V600-Mutant Metastatic Melanoma
