# Supplementary material for: Longitudinal Genomic Analysis to Fine-tune Targeted Therapy: Results of the Phase II LOGIC 2 Trial in Patients with BRAFV600-Mutant Metastatic Melanoma
Source: Clin Cancer Res. 2025 Mar 19;31(11):2097–107. doi: 10.1158/1078-0432.CCR-24-0254 (PMC12130804; doi:10.1158/1078-0432.CCR-24-0254)
Supplement: Supplementary Data 1 — Longitudinal Genomic Analysis to Fine-Tune Targeted Therapy: Results of the Phase II LOGIC 2 Trial in Patients With BRAF V600-Mutant Metastatic Melanoma [file ccr-24-0254_supplementary_data_1_suppds1.pdf]

SUPPLEMENTARY DATA

Longitudinal Genomic Analysis to Fine-Tune Targeted Therapy: Results of the Phase II LOGIC 2 Trial in Patients With BRAF V600-Mutant Metastatic Melanoma

Authors: Reinhard Dummer[1], Shahneen Sandhu[2], Wilson H. Miller, Jr[3], Marcus O. Butler[4], Matthew H. Taylor[5], Lucie Heinzerling[6], Christian U. Blank[7], Eva Muñoz Couselo[8], Howard A. Burris 3rd[9], Michael A. Postow[10], Bartosz Chmielowski[11], Mark R. Middleton[12], Carola Berking[13], Jessica C. Hassel[14], Anja Heike Gesierich[15], Cornelia Mauch[16], Joseph F. Kleha[17], Anna Polli[18], Allison S. Harney[19], Alessandra di Pietro[18], Paolo A. Ascierto[20]

Affiliations: [1]Department of Dermatology, Skin Cancer Unit, University Hospital Zurich, Zurich, Switzerland; [2]Sir Peter MacCallum Cancer Department of Oncology, University of Melbourne, Melbourne, Australia; [3]Lady Davis Institute and Segal Cancer Centre, Jewish General Hospital, Departments of Medicine and Oncology, McGill University, Montreal, Canada; [4]Princess Margaret Cancer Centre, University Health Network, Departments of Medicine and Immunology, University of Toronto, Toronto, Canada; [5]Earle A. Chiles Research Institute, Providence Cancer Institute, Portland, OR; [6]Department of Dermatology and Allergy, University Hospital, Ludwig Maximilian University, Munich, Germany; [7]Department of Medical Oncology, Netherlands Cancer Institute, Amsterdam, the Netherlands; [8]Department of Medical Oncology, Melanoma and Other Skin Cancers Unit, Vall d'Hebron Hospital and Vall d'Hebron Institute of Oncology (VHIO), Barcelona, Spain; [9]Sarah Cannon Research Institute, Nashville, TN; [10]Department of Medicine, Memorial Sloan Kettering Cancer Center and Weill Cornell Medical College, New York, NY; [11]Jonsson Comprehensive Cancer Center, Division of Hematology-Oncology, Department of Medicine, University of California Los Angeles, Los Angeles, CA, USA; [12]NIHR Oxford Biomedical Research Centre, Oxford University Hospitals NHS Foundation Trust, John Radcliffe Hospital, Oxford, UK; Department of Oncology, University of Oxford, Oxford, UK; Early Phase Clinical Trials Unit, Cancer & Haematology Centre, Churchill Hospital, Oxford, UK; [13]Department of Dermatology, Uniklinikum Erlangen, CCC Erlangen – EMN, Friedrich-Alexander-University Erlangen-Nürnberg (FAU), Erlangen, Germany; [14]Heidelberg University, Medical Faculty Heidelberg, Department of Dermatology and National Center for Tumor Diseases (NCT), NCT Heidelberg, a partnership between DKFZ and University Hospital Heidelberg, Heidelberg, Germany; [15]Department of Dermatology, Venerology and Allergology, University Hospital Würzburg, Würzburg, Germany, [16]Department of Dermatology and Venereology, Faculty of Medicine and University Hospital of Cologne, Cologne, Germany; [17]Pfizer, New York, NY; [18]Pfizer, Milan, Italy; [19]Pfizer Boulder Research Unit, Boulder, CO; [20]Melanoma, Cancer Immunotherapy and Innovative Therapies Unit, Istituto Nazionale Tumori IRCCS Fondazione Pascale, Napoli, Italy

Corresponding Author: Reinhard Dummer, Department of Dermatology, University Hospital Zurich, Zurich, Switzerland. Email: reinhard.dummer@usz.ch

Supplementary Fig. S1. Waterfall plot of percentage change from baseline in target lesions as per local assessment in (A) Part I/Run-in naive patients, (B) Part I/Run-in pretreated patients, (C) Part II encorafenib plus binimetinib and ribociclib, (D) Part II encorafenib plus binimetinib and capmatinib, and (E) Part II encorafenib plus binimetinib and buparlisib. n is the total number of subjects with best percentage change from baseline in the sum of the longest target lesion diameters as per local assessment. N is the total number of subjects in full analysis set.

(A)

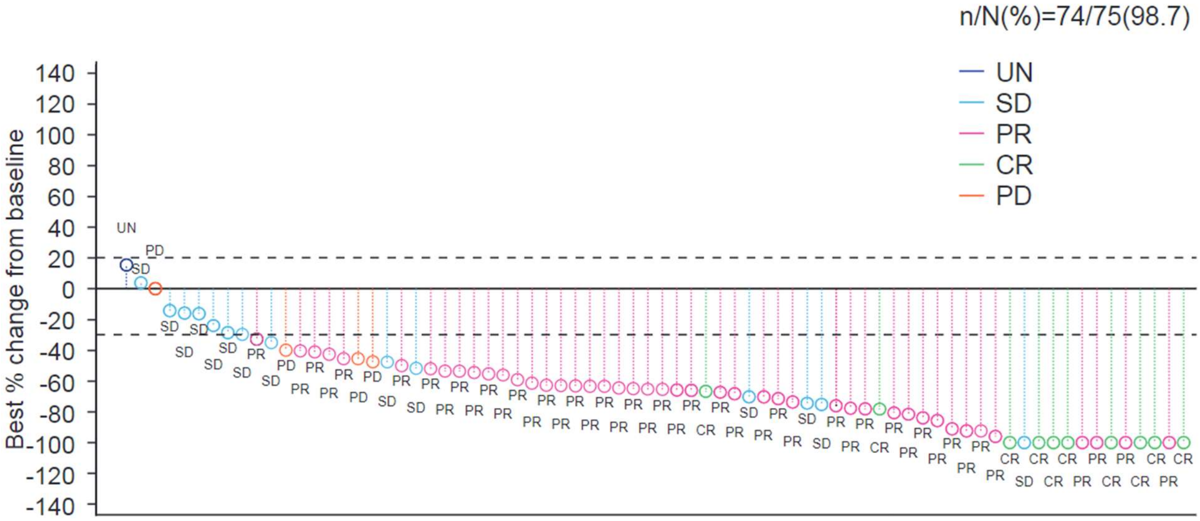

(B)

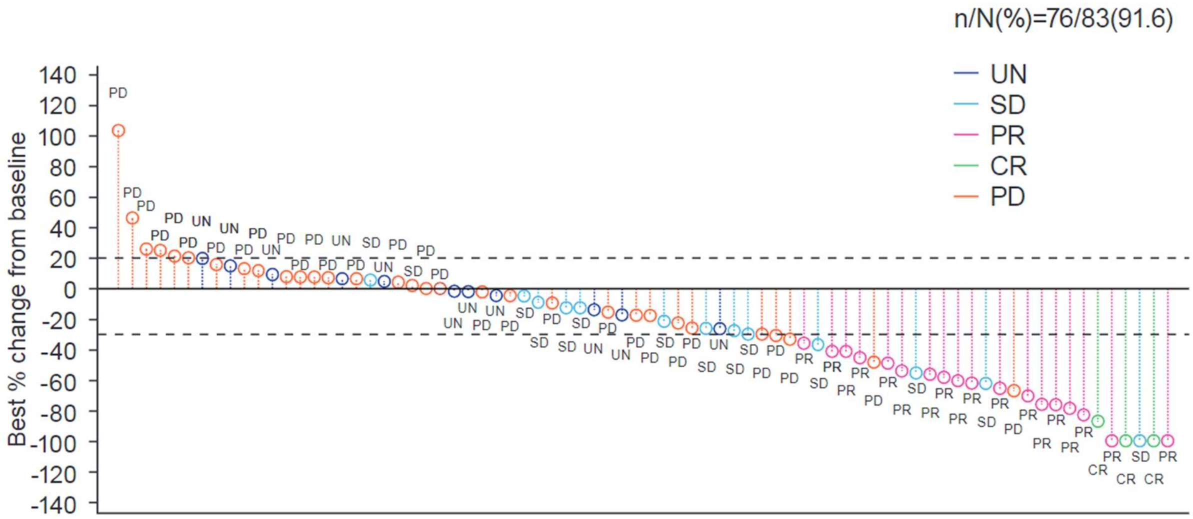

(C)

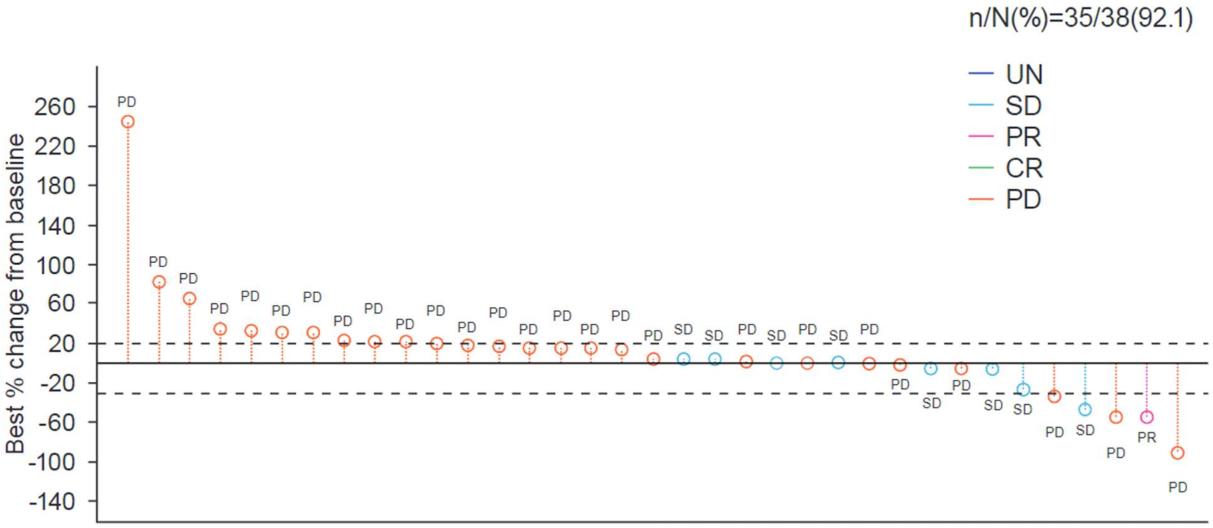
n/N(%)=35/38(92.1)
UN
SD
PR
CR
PD
Best % change from baseline
260
220
180
140
100
60
20
-20
-60
-100
-140

(D)

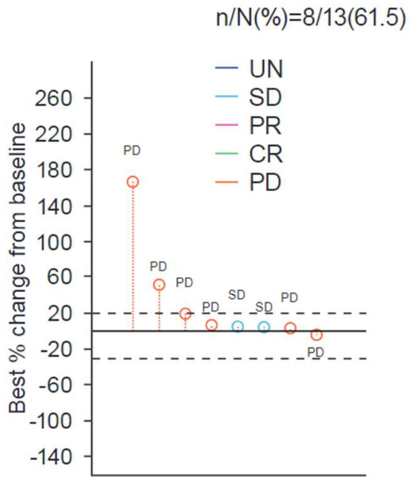
n/N(%)=8/13(61.5)
UN
SD
PR
CR
PD
Best % change from baseline
260
220
180
140
100
60
20
-20
-60
-100
-140
PD
PD
PD
PD
SD
SD
PD
PD

(E)

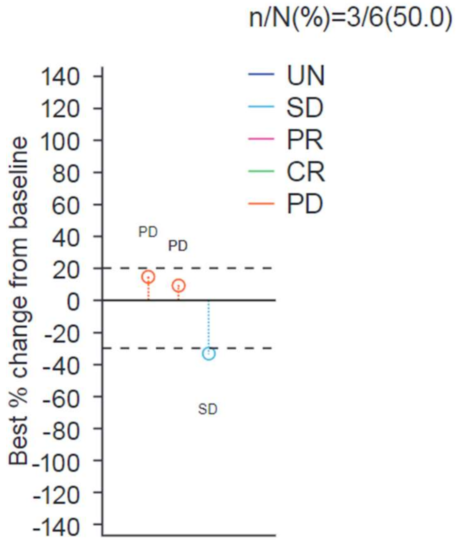
n/N(%)=3/6(50.0)
UN
SD
PR
CR
PD
Best % change from baseline
140
120
100
80
60
40
20
0
-20
-40
-60
-80
-100
-120
-140
PD
PD
SD

Supplementary Fig. S2. TTR per local assessment (FAS population; Part II, confirmed responders). Kaplan-Meier analysis of time to response in the encorafenib plus binimetinib and ribociclib, encorafenib plus binimetinib and infigratinib, encorafenib plus binimetinib and capmatinib, and encorafenib plus binimetinib and buparlisib arms. CI, confidence interval; FAS, full analysis set; NE, not evaluable; TTR, time to response.

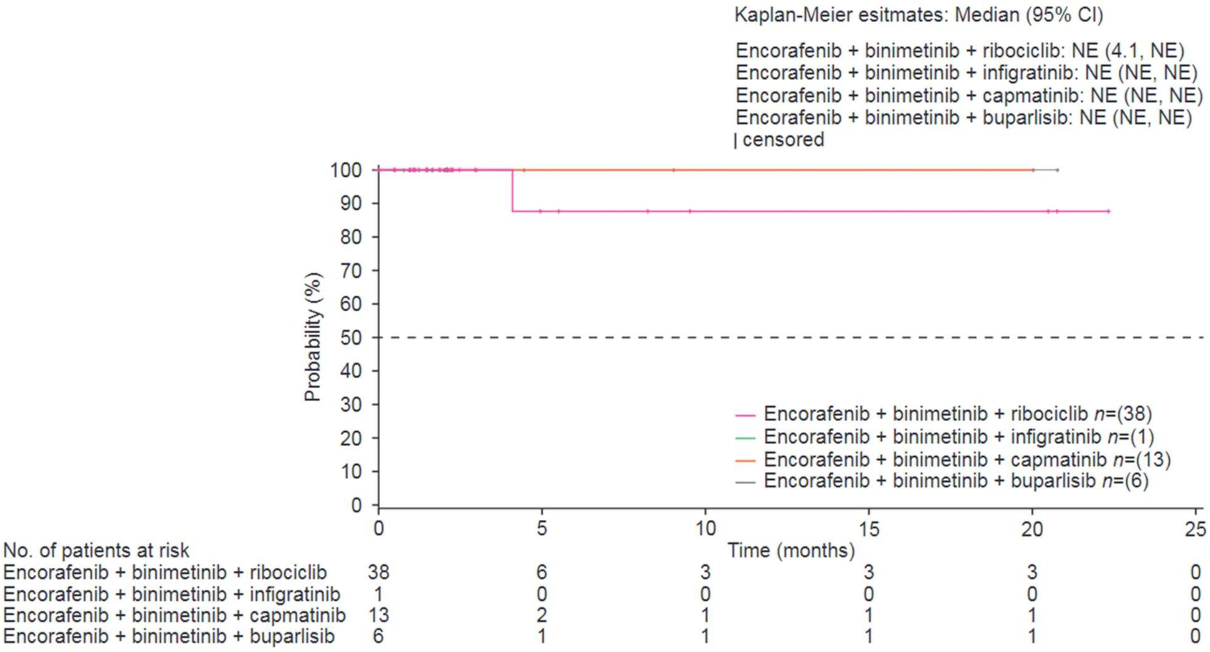

Supplementary Table S1. Baseline demographics and disease characteristics in Part I/Run-in (FAS population)

	**Encorafenib + binimetinib (naive)** ***n*=75**	**Encorafenib + binimetinib (pretreated)** ***n*=83**
**Median age (range), years**	56.0 (23-80)	53.9 (29-83)
**Sex, *n* (%)**		
Male	47 (62.7)	44 (53.0)
Female	28 (37.3)	39 (47.0)
**Race, *n* (%)**		
White	74 (98.7)	82 (98.8)
Asian	1 (1.3)	1 (1.2)
**Ethnicity, *n* (%)**		
Not Hispanic or Latino	72 (96.0)	76 (91.6)
Hispanic or Latino	3 (4.0)	7 (8.4)
**ECOG PS, *n* (%)**		
0	55 (73.3)	41 (49.4)
1	19 (25.3)	35 (42.2)
2	1 (1.3)	5 (6.0)
Missing	0	2 (2.4)
**Stage at time of study entry, *n* (%)**		
IIIC	5 (6.7)	1 (1.2)
IV	60 (80.0)	71 (85.5)
IVA	2 (2.7)	1 (1.2)

IVB	8 (10.7)	9 (10.8)
Missing	0	1 (1.2)
**Prior antineoplastic therapy received, *n* (%)**	30 (40.0)	83 (100)
**Time between end of last medication and start of treatment, median (range), days**	71.5 (17–5977)	29.0 (1–268)
**Prior antineoplastic therapy by ATC class, *n* (%)**		
Anthracyclines and related substances	0	1 (1.2)
Antineoplastic agents	0	1 (1.2)
Aromatase inhibitors	0	1 (1.2)
Imidazothiazole derivatives	1 (1.3)	0
Immunostimulants	0	3 (3.6)
Interferons	12 (16.0)	17 (20.5)
Interleukins	1 (1.3)	1 (1.2)
Investigational drug	0	2 (2.4)
Monoclonal antibodies	21 (28.0)	49 (59.0)
Nitrogen mustard analogues	0	2 (2.4)
Other alkylating agents	2 (2.7)	12 (14.5)
Other cytotoxic antibiotics	1 (1.3)	1 (1.2)
Other immunostimulants	1 (1.3)	1 (1.2)
Platinum compounds	0	8 (9.6)
Podophyllotoxin derivatives	0	1 (1.2)
Protein kinase inhibitors[a]	0	83 (100)
Purine analogues	0	1 (1.2)
Taxanes	0	7 (8.4)

Uncoded	0	1 (1.2)
Vinca alkaloids and analogues	0	1 (1.2)

[a]Protein kinase inhibitors were only used in the pretreated arm and included dabrafenib (n=43), vemurafenib (n=36), trametinib (n=30), encorafenib (n=18), binimetinib (n=5), cobimetinib (n=2), and dabrafenib mesylate (n=1).

ATC, Anatomical Therapeutic Chemical, ECOG PS, Eastern Cooperative Oncology Group performance status; FAS, full analysis set.

Supplementary Table S2. Baseline demographics and disease characteristics for Part II (FAS population)

	**Encorafenib + binimetinib + ribociclib *n*=38**	**Encorafenib + binimetinib + infigratinib *n*=1**	**Encorafenib + binimetinib + capmatinib *n*=13**	**Encorafenib + binimetinib + buparlisib *n*=6**
**Median age (range), years**	56.0 (24-82)	62.0 (62-62)	62.0 (48-80)	49.5 (34-66)
**Sex, *n* (%)**				
Male	16 (42.1)	0	8 (61.5)	5 (83.3)
Female	22 (57.9)	1 (100)	5 (38.5)	1 (16.7)
**Race, *n* (%)**				
White	37 (97.4)	1 (100)	13 (100)	6 (100)
Asian	1 (2.6)	0	0	0
**Ethnicity, *n* (%)**				
Not Hispanic or Latino	37 (97.4)	1 (100)	13 (100)	6 (100)
Hispanic or Latino	1 (2.6)	0	0	0
**ECOG PS, *n* (%)**				
0	17 (44.7)	1 (100)	11 (84.6)	3 (50.0)
1	20 (52.6)	0	2 (15.4)	2 (33.3)
2	1 (2.6)	0	0	1 (16.7)
**Stage at time of study entry, *n* (%)**				

IIIC	3 (7.9)	0	0	1 (16.7)
IV	30 (78.9)	1 (100)	11 (84.6)	4 (66.7)
IVA	1 (2.6)	0	0	0
IVB	4 (10.5)	0	2 (15.4)	1 (16.7)
**Prior antineoplastic therapy received, *n* (%)**	30 (78.9)	1 (100)	10 (76.9)	5 (83.3)
**Time between end of last medication and start of triple combination treatment, median (range), days**	28.5 (1–346)	268.0 (268–268)	68.0 (7–105)	14.0 (7–205)
**Prior antineoplastic therapy by ATC class, *n* (%)**				
Immunostimulants	2 (5.3)	0	0	0
Interferons	5 (13.2)	0	3 (23.1)	1 (16.7)
Monoclonal antibodies	18 (47.4)	1 (100)	5 (38.5)	3 (50.0)
Other alkylating agents	6 (15.8)	0	0	0
Other cytotoxic antibiotics	0	1 (100)	1 (7.7)	0
Other immunostimulants	1 (2.6)	0	0	0
Platinum compounds	3 (7.9)	0	0	0
Protein kinase inhibitors[a]	25 (65.8)	1 (100)	7 (53.8)	5 (83.3)
Taxanes	3 (7.9)	0	0	0

[a]Protein kinase inhibitors included dabrafenib (n=12 in the ribociclib arm, n=1 in the infigratinib arm, n=1 in the capmatinib arm, and n=4 in the buparlisib arm); trametinib (n=13 in the ribociclib arm, n=1 in the infigratinib arm, n=2 in the capmatinib arm, and n=1 in the buparlisib arm);

vemurafenib (n=9 in the ribociclib arm, n=0 in the infigratinib arm, n=4 in the capmatinib arm, and n=2 in the buparlisib arm); encorafenib (n=7 in the ribociclib arm, n=0 in the infigratinib arm, n=1 in the capmatinib arm, and n=1 in the buparlisib arm); binimetinib (n=5 in the ribociclib arm, n=0 each in the infigratinib, capmatinib, and buparlisib arms); and dabrafenib mesylate (n=1 in the ribociclib arm, n=0 each in the infigratinib, capmatinib, and buparlisib arms).

ATC, Anatomical Therapeutic Chemical, ECOG PS, Eastern Cooperative Oncology Group performance status; FAS, full analysis set.

Supplementary Table S3. Representativeness of study participants

Cancer type	Melanoma
Considerations related to:	
Sex and gender	Globally in 2020, 53.6% of the total new cases of melanoma occurred in males.
Age	The average age at melanoma diagnosis is 66 years old, however, melanoma is one of the most common cancers in young adults.
Race or ethnicity	Melanoma is more frequent in White people, although POC experience delayed diagnosis and higher mortality.
Geography	Most new cases of melanoma occur in North America, Europe, and Australia/New Zealand.
Overall representativeness of this study	In our study, there was a majority of males (Part I/Run-in: 57.6% male; Part II: 50.0% male) in total and most patients were White (>90% in both parts of the study) and Not Hispanic or Latino (>90% in both parts of the study). The range of median ages across all arms was 53.0–56.0 years old (range 23–83 years) in Part I/Run-in and 49.5–62.0 years old (range 24–82 years) in Part II. LOGIC 2 was a global study with sites in Australia, Europe, and North America.

Supplementary Table S4. Prior anticancer therapies (Part I/Run-in)

n (%)	**Encorafenib + binimetinib (naive) *n*=75**	**Encorafenib + binimetinib (pretreated) *n*=83**
**Surgery**	74 (98.7)	79 (95.2)
**Radiotherapy**	18 (24.0)	39 (47.0)
**Medication**[a]	30 (40.0)	83 (100)
Anthracyclines and related substances	0	1 (1.2)
Antineoplastic agents	0	1 (1.2)
Aromatase inhibitors	0	1 (1.2)
Imidazothiazole derivatives	1 (1.3)	0
Immunostimulants[b]	0	3 (3.6)
Interferons	12 (16.0)	17 (20.5)
Interleukins	1 (1.3)	1 (1.2)
Investigational drug	0	2 (2.4)
Monoclonal antibodies	21 (28.0)	49 (59.0)
Nitrogen mustard analogues	0	2 (2.4)
Other alkylating agents	2 (2.7)	12 (14.5)
Other cytotoxic antibiotics	1 (1.3)	1 (1.2)
Other immunostimulants	1 (1.3)	1 (1.2)
Platinum compounds	0	8 (9.6)
Podophyllotoxin derivatives	0	1 (1.2)
Protein kinase inhibitors	0	83 (100)
Purine analogues	0	1 (1.2)

Taxanes	0	7 (8.4)
Uncoded	0	1 (1.2)
Vinca alkaloids and analogues	0	1 (1.2)

[a]By anatomical therapeutic chemical classification.

[b]Immunostimulants consisted of anti-tumor necrosis factor monoclonal antibody.

Supplementary Table S5. Genomic alterations at baseline and end of treatment (Part I/Run-in)

n (%)	Baseline		End of treatment	
	**Encorafenib + binimetinib (naive) *n*=75**	**Encorafenib + binimetinib (pretreated) *n*=83**	**Encorafenib + binimetinib (naive) *n*=75**	**Encorafenib + binimetinib (pretreated) *n*=83**
BRAF				
Copy number variant/ratio, mean (standard deviation)	2.2 (1.0)	2.4 (1.2)	2.4 (0.8)	10.1 (12.9)
Rearrangement/genomic position	0	9 (10.8)	8 (10.7)	11 (13.3)
V600E	46 (61.3)	53 (63.9)	18 (24.0)	19 (22.9)
V600G	1 (1.3)	0	0	0
V600K	4 (5.3)	7 (8.4)	0	4 (4.8)
V600R	1 (1.3)	0	1 (1.3)	0
MET				
Copy number variant/ratio, mean (standard deviation)	2.1 (0.8)	1.8 (0.4)	4.3 (2.5)	4.3 (4.1)
Rearrangement/genomic position	0	0	1 (1.3)	0
PTEN				
Copy number variant/ratio, mean (standard deviation)	0.2 (0.1)	0.4 (0.2)	0.4 (0.3)	0.3 (0.2)

Rearrangement/genomic position	0	0	0	1 (1.2)

Supplementary Table S6. Gene alterations for enrollment in Part II

	**Gene**	**Total, *n***	**Mutations (amino acid changes, *n*)**	**Amplification,[b] *n***	**Loss of copy number,[c] *n***
Encorafenib + binimetinib + ribociclib	*KRAS*	1	A146V, 1		
	NRAS	6	Q61R, 2<br>Q61K, 4[a]		
	HRAS	1	G13R, 1		
	CDKN2A	13	splice site 151-1G>A, 2<br>V126D, 1<br>D146fs*12+, 1<br>E61*, 1<br>Y44fs*1, 1		7
	BRAF	2		2	
	CDK4	1	R24H, 1		
	MAP2K1	1	F531, 1		
Encorafenib + binimetinib + buparlisib	*PTEN*	1			1
	PIK3CA	1	M1043V, 1		
Encorafenib + binimetinib + capmatinib	*MET*	2		2	

[a]One patient had both Q61R and Q61K.

[b]Copy number ratio >1.

[c]Copy number ratio <1.

Supplementary Table S7. New mutations identified at progression in Part II

Gene	Total, *n*	Mutations (amino acid changes, *n*)	Rearrangement, *n*	Amplification,[a] *n*	Loss of copy number,[b] *n*
BRAF	11	V600E, 3<br>V600K, 1	5	4	
CDKN2A	8	L64_H66del, 1			7
H/N/KRAS	8	*KRAS* A146V, 1<br>*NRAS* Q61R, 2<br>*NRAS* Q61K, 4<br>*HRAS* G13R, 1			
TERT promoter	6	-146C>T, 5<br>-124C>T, 1			
CDKN2B	5				5
PTEN	4			4	
CDK6	3			3	
HGF	3			3	
KEL	3			3	

[a]Copy number ratio >1.

[b]Copy number ratio <1.

Supplementary Table S8. Duration of exposure (safety set; Part I/Run-in)

**Duration of exposure in weeks**	**Encorafenib + binimetinib (naive)** ***n*=75**	**Encorafenib + binimetinib (pretreated)** ***n*=83**
Exposure categories, *n* (%)		
<4	1 (1.3)	4 (4.8)
4 to <8	1 (1.3)	23 (27.7)
8 to <12	1 (1.3)	11 (13.3)
12 to <24	9 (12.0)	17 (20.5)
24 to <36	13 (17.3)	9 (10.8)
36 to <48	14 (18.7)	5 (6.0)
48 to <60	5 (6.7)	3 (3.6)
60 to <72	4 (5.3)	0
72 to <84	2 (2.7)	2 (2.4)
84 to < 96	2 (2.7)	1 (1.2)
96 to <108	2 (2.7)	0
≥108	21 (28.0)	8 (9.6)
Exposure, median (range), weeks	47.6 (3.9-403.7)	13.0 (0.1-385.3)

Supplementary Table S9. Duration of exposure (safety set; Part II)

**Duration of exposure in weeks**	**Encorafenib + binimetinib + ribociclib *n*=38**	**Encorafenib + binimetinib + infigratinib *n*=1**	**Encorafenib + binimetinib + capmatinib *n*=13**	**Encorafenib + binimetinib + buparlisib *n*=6**
Exposure categories, *n* (%)				
<4	6 (15.8)	0	1 (7.7)	1 (16.7)
4 to <8	9 (23.7)	0	5 (38.5)	3 (50.0)
8 to <12	13 (34.2)	1 (100)	3 (23.1)	0
12 to <24	3 (7.9)	0	2 (15.4)	2 (33.3)
≥24	7 (18.4)	0	2 (15.4)	0
Exposure, median (range), weeks	9.7 (0.9-97.0)	9.4	9.1 (3.0-38.7)	5.0 (0.9-14.9)

Supplementary Table S10. AEs suspected of being study drug related occurring in >10% of patients (safety set; Part I/Run-in)

The most frequently reported AEs suspected to be study drug related in Part I/Run-in were nausea (31.6%), increased blood creatine phosphokinase (26.6%), diarrhea (24.1%), fatigue (24.1%), retinopathy (19.6%), vomiting (17.7%), arthralgia (15.2%), constipation (10.8%), and blurred vision (10.8%).

**Preferred term, *n* (%)**	**Encorafenib + binimetinib[a] (naive) *n*=75**	**Encorafenib + binimetinib[a] (pretreated) *n*=83**
Total	71 (94.7)	64 (77.1)
Nausea	23 (30.7)	27 (32.5)
Blood creatine phosphokinase increase	31 (41.3)	11 (13.3)
Diarrhea	23 (30.7)	15 (18.1)
Fatigue	22 (29.3)	16 (19.3)
Retinopathy	21 (28.0)	10 (12.0)
Vomiting	8 (10.7)	20 (24.1)
Arthralgia	14 (18.7)	10 (12.0)
Constipation	12 (16.0)	5 (6.0)
Vision blurred	10 (13.3)	7 (8.4)
Alanine aminotransferase increase	10 (13.3)	6 (7.2)
Dry skin	6 (8.0)	10 (12.0)

Gamma-glutamyl transferase increase	9 (12.0)	7 (8.4)
Lipase increase	8 (10.7)	8 (9.6)
Pyrexia	4 (5.3)	12 (4.5)
Rash	5 (6.7)	11 (13.3)
Abdominal pain	9 (12.0)	5 (6.0)
Myalgia	10 (13.3)	4 (4.8)
Alopecia	9 (12.0)	4 (4.8)
Muscle spasms	10 (13.3)	3 (3.6)
Edema peripheral	9 (12.0)	4 (4.8)
Anemia	8 (10.7)	4 (4.8)

[a]Only AEs occurring during encorafenib/binimetinib combination treatment or within 30 days of the last study encorafenib/binimetinib combination treatment were reported.

AE, adverse event.

Supplementary Table S11. SAEs regardless of study treatment (safety set; Part I/Run-in)

The most frequently reported Grade 3/4 AEs in Part I/Run-in were anemia (8.0%), increased blood creatine phosphokinase (8.0%), increased alanine aminotransferase (6.7%), gamma-glutamyl transferase increase (6.7%), increased lipase (6.7%), nausea (5.3%), diarrhea (5.3%), epilepsy (4.0%), and vomiting (4.0%) in naive patients, and anemia (6.0%), nausea (4.8%), general physical health deterioration (4.8%), increased lipase (4.8%), and arthralgia (4.8%) in pretreated patients.

**Preferred term, *n* (%)**	**Encorafenib + binimetinib[a] (naive) *n*=75**	**Encorafenib + binimetinib[a] (pretreated) *n*=83**
Vomiting	2 (2.7)	6 (7.2)
Nausea	4 (5.3)	3 (3.6)
Diarrhea	4 (5.3)	1 (1.2)
Epilepsy	3 (4.0)	2 (2.4)
General physical health deterioration	1 (1.3)	4 (4.8)
Anemia	3 (4.0)	1 (1.2)
Pneumonia	4 (5.3)	0
Pyrexia	0	4 (4.8)
Sepsis	1 (1.3)	3 (3.6)
Terminal ileitis	2 (2.7)	1 (1.2)
Abdominal pain	1 (1.3)	1 (1.2)

Blood creatine phosphokinase increase	1 (1.3)	1 (1.2)
Dehydration	0	2 (2.4)
Delirium	1 (1.3)	1 (1.2)
Diverticulitis	2 (2.7)	0
Fatigue	1 (1.3)	1 (1.2)
Gastric ulcer	1 (1.3)	1 (1.2)
Hematuria	2 (2.7)	0
Lipase increase	0	2 (2.4)
Partial seizures	2 (2.7)	0
Rectal hemorrhage	1 (1.3)	1 (1.2)
Seizure	2 (2.7)	0
Tumor pain	0	2 (2.4)
Urinary tract infection	1 (1.3)	1 (1.2)
Urosepsis	2 (2.7)	0
Acute kidney injury	0	1 (1.2)
Acute myocardial infarction	1 (1.3)	0
Adrenomegaly	1 (1.3)	0
Angioedema	1 (1.3)	0
Aphasia	1 (1.3)	0
Appendicitis	1 (1.3)	0
Arthritis	0	1 (1.2)

Arthritis bacterial	1 (1.3)	0
Aspartate aminotransferase increase	0	1 (1.2)
Asthenia	1 (1.3)	0
Back pain	1 (1.3)	0
Basal cell Carcinoma	0	1 (1.2)
Benign prostatic hyperplasia	1 (1.3)	0
Bone pain	1 (1.3)	0
Brain edema	1 (1.3)	0
Breast cancer	1 (1.3)	0
Breast cancer Recurrent	1 (1.3)	0
Cancer pain	0	1 (1.2)
Cerebral hemorrhage	1 (1.3)	0
Cerebrovascular accident	1 (1.3)	0
Chills	0	1 (1.2)
Chorioretinitis	1 (1.3)	0
Chronic obstructive pulmonary disease	1 (1.3)	0

Clostridium difficile colitis	1 (1.3)	0
Colitis	0	1 (1.2)
Constipation	0	1 (1.2)
Costochondritis	1 (1.3)	0
Decreased appetite	0	1 (1.2)
Device related infection	1 (1.3)	0
Disease progression	1 (1.3)	0
Drug eruption	0	1 (1.2)
Dysphagia	1 (1.3)	0
Dyspnea	0	1 (1.2)
Ejection fraction decrease	1 (1.3)	0
Epistaxis	0	1 (1.2)
Erysipelas	0	1 (1.2)
Facial bones fracture	1 (1.3)	0
Fall	0	1 (1.2)
Febrile infection	0	1 (1.2)
Fibromyalgia	1 (1.3)	0
Headache	1 (1.3)	0
Hemiparesis	1 (1.3)	0
Hemiplegia	1 (1.3)	0

Hiccups	0	1 (1.2)
Hypotension	0	1 (1.2)
Inguinal hernia	0	1 (1.2)
Intestinal mass	0	1 (1.2)
Intraocular pressure increase	1 (1.3)	0
Intussusception	1 (1.3)	0
Metastases to bone	1 (1.3)	0
Metastases to spine	0	1 (1.2)
Metastatic malignant melanoma	1 (1.3)	0
Musculoskeletal pain	0	1 (1.2)
Myalgia	1 (1.3)	0
Myocardial infarction	1 (1.3)	0
Myopathy toxic	1 (1.3)	0
Nephrolithiasis	1 (1.3)	0
Non-cardiac chest pain	1 (1.3)	0
Osteoradionecrosis	1 (1.3)	0
Pain	1 (1.3)	0
Pain in extremity	1 (1.3)	0
Paresthesia	1 (1.3)	0
Paresis	1 (1.3)	0

Peripheral sensory neuropathy	1 (1.3)	0
Periprosthetic fracture	1 (1.3)	0
Pneumothorax	0	1 (1.2)
Post procedural hemorrhage	0	1 (1.2)
Procedural pneumothorax	0	1 (1.2)
Prostate cancer	0	1 (1.2)
Pulmonary alveolar hemorrhage	0	1 (1.2)
Pyelonephritis	0	1 (1.2)
Renal cell carcinoma	1 (1.3)	0
Seroma	0	1 (1.2)
Shock	0	1 (1.2)
Sinus tachycardia	0	1 (1.2)
Spinal cord compression	0	1 (1.2)
Streptococcal infection	1 (1.3)	0
Subileus	0	1 (1.2)
Thrombocytopenia	1 (1.3)	0
Troponin T increase	1 (1.3)	0

Urticaria	0	1 (1.2)
Vasculitis	1 (1.3)	0
Vertigo	1 (1.3)	0
Vertigo positional	1 (1.3)	0
Vestibular neuronitis	1 (1.3)	0
Vision blurred	1 (1.3)	0
Wrist fracture	1 (1.3)	0

[a]Only AEs occurring during treatment or within 30 days of the last study medication were reported.

AE, adverse event; SAE, serious adverse event.

Supplementary Table S12. Overview of dose reductions (safety set, Part I/Run-in)

	**Encorafenib + binimetinib[a] (naive) *n*=75**		**Encorafenib + binimetinib[a] (pretreated) *n*=83**	
	**Encorafenib *n* (%)**	**Binimetinib *n* (%)**	**Encorafenib *n* (%)**	**Binimetinib *n* (%)**
Without dose reduction	66 (88.0)	43 (57.3)	69 (83.1)	55 (66.3)
With at least one dose reduction	9 (12.0)	32 (42.7)	14 (16.9)	28 (33.7)
With only one dose reduction	4 (5.3)	5 (6.7)	5 (6.0)	9 (10.8)
With more than one dose reduction	5 (6.7)	27 (36.0)	9 (10.8)	19 (22.9)
With at least one dose reduction by reason				
As per protocol	4 (5.3)	7 (9.3)	1 (1.2)	3 (3.6)
AE	5 (6.7)	24 (32.0)	9 (10.8)	20 (24.1)

Disease progression	0	3 (4.0)	0	3 (3.6)
Dispensing error	1 (1.3)	1 (1.3)	2 (2.4)	0
Dosing error	1 (1.3)	13 (17.3)	2 (2.4)	10 (12.0)
Physician decision	1 (1.3)	9 (12.0)	2 (2.4)	4 (4.8)
Protocol deviation	0	0	0	2 (2.4)
Patient/guardian decision	1 (1.3)	6 (8.0)	1 (1.2)	3 (3.6)
Technical problems	0	4 (5.3)	0	2 (2.4)

[a]A patient with multiple dose reductions was only counted once in each dose reduction category. Each patient could have multiple reasons for dose reduction.

AE, adverse event.

Supplementary Table S13. Overview of dose interruptions (safety set; Part I/Run-in)

	**Encorafenib + binimetinib[a] (naive) *n*=75**		**Encorafenib + binimetinib[a] (pretreated) *n*=83**	
	**Encorafenib *n* (%)**	**Binimetinib *n* (%)**	**Encorafenib *n* (%)**	**Binimetinib *n* (%)**
Without dose interruption	29 (38.7)	31 (41.3)	43 (51.8)	42 (50.6)
With at least one dose interruption	46 (61.3)	44 (58.7)	40 (48.2)	41 (49.4)
With only one dose interruption	17 (22.7)	19 (25.3)	22 (26.5)	23 (27.7)
With more than one dose interruption	29 (38.7)	25 (33.3)	18 (21.7)	18 (21.7)
With at least one dose interruption by reason				
AE	39 (52.0)	39 (52.0)	32 (38.6)	33 (39.8)

Disease progression	1 (1.3)	1 (1.3)	0	0
Dispensing error	0	1 (1.3)	1 (1.2)	1 (1.2)
Dosing error	10 (13.3)	7 (9.3)	7 (8.4)	5 (6.0)
Physician decision	11 (14.7)	10 (13.3)	5 (6.0)	6 (7.2)
Protocol deviation	1 (1.3)	0	1 (1.2)	0
Patient/guardian decision	6 (8.0)	5 (6.7)	6 (7.2)	4 (4.8)
Technical problems	5 (6.7)	3 (4.0)	0	0

[a]A patient with multiple dose interruptions was only counted once in each category. Each patient could have multiple reasons for dose interruptions.

AE, adverse event.

Supplementary Table S14. AEs, suspected to be study drug related, occurring in >10% of patients (safety set; Part II)

The most frequently reported AEs suspected to be study drug related in Part I/Run-in were nausea (28.9%) and diarrhea (21.1%) in the encorafenib plus binimetinib and ribociclib arm, peripheral edema (23.1%) in the encorafenib plus binimetinib and capmatinib arm, and increased aspartate aminotransferase (33.3%) in the encorafenib plus binimetinib and buparlisib arm.

**Preferred term, *n* (%)**	**Encorafenib + binimetinib + ribociclib[a] *n*=38**	**Encorafenib + binimetinib + infigratinib[a] *n*=1**	**Encorafenib + binimetinib + capmatinib[a] *n*=13**	**Encorafenib + binimetinib + buparlisib[a] *n*=6**
Total	32 (84.2)	0	10 (76.9)	3 (50.0)
Nausea	11 (28.9)	0	2 (15.4)	1 (16.7)
Diarrhea	8 (21.1)	0	0	0
Vomiting	7 (18.4)	0	0	1 (16.7)
Fatigue	6 (15.8)	0	1 (7.7)	0
Anemia	5 (13.2)	0	1 (7.7)	1 (16.7)
Blood creatine phosphokinase increase	5 (13.2)	0	2 (15.4)	0

Alanine aminotransferase increase	4 (10.5)	0	1 (7.7)	1 (16.7)
Pyrexia	4 (10.5)	0	0	0
White blood cell count decrease	4 (10.5)	0	0	0
Aspartate aminotransferase increase	3 (7.9)	0	0	2 (33.3)
Blood creatinine increase	2 (5.3)	0	2 (15.4)	0
Gamma-glutamyl transferase increase	2 (5.3)	0	2 (15.4)	1 (16.7)
Edema peripheral	2 (5.3)	0	3 (23.1)	0
Abdominal pain	1 (2.6)	0	0	1 (16.7)
Dysgeusia	0	0	0	1 (16.7)
Hypertension	0	0	1 (7.7)	1 (16.7)
Hypoalbuminemia	0	0	2 (15.4)	0

Hypothyroidism	0	0	0	1 (16.7)

[a]Only AEs occurring during triplet combination treatment or within 30 days of the last study treatment are reported.

AE, adverse event.

Supplementary Table S15. SAEs regardless of study treatment relationship (safety set; Part II)

Preferred term *n* (%)	Encorafenib + binimetinib + ribociclib[a] *n*=38	Encorafenib + binimetinib + infigratinib[a] *n*=1	Encorafenib + binimetinib + capmatinib[a] *n*=13	Encorafenib + binimetinib + buparlisib[a] *n*=6
Total	19 (50.0)	0	6 (46.2)	4 (66.7)
Anemia	2 (5.3)	0	1 (7.7)	0
Adrenocortical insufficiency acute	1 (2.6)	0	0	0
Alanine aminotransferase increase	1 (2.6)	0	0	0
Aspartate aminotransferase increase	1 (2.6)	0	0	0
Back pain	1 (2.6)	0	0	1 (16.7)
Cancer pain	1 (2.6)	0	1 (7.7)	0
Colitis ulcerative	1 (2.6)	0	0	0
Constipation	1 (2.6)	0	0	0

Dehydration	1 (2.6)	0	0	0
Diplopia	1 (2.6)	0	0	0
Embolic cerebral infarction	1 (2.6)	0	0	0
Exophthalmos	1 (2.6)	0	0	0
General physical health deterioration	1 (2.6)	0	0	0
Groin pain	1 (2.6)	0	0	0
Hypoesthesia	1 (2.6)	0	0	0
Nausea	1 (2.6)	0	1 (7.7)	0
Pain	1 (2.6)	0	0	0
Pain in extremity	1 (2.6)	0	0	1 (16.7)
Paraparesis	1 (2.6)	0	0	0
Pathological fracture	1 (2.6)	0	0	0
Pneumonia	1 (2.6)	0	0	0
Pulmonary embolism	1 (2.6)	0	0	0
Pyrexia	1 (2.6)	0	1 (7.7)	0

Syncope	1 (2.6)	0	0	0
Tumor ulceration	1 (2.6)	0	0	0
Vomiting	1 (2.6)	0	0	0
Wound infection	1 (2.6)	0	0	0
Aphasia	0	0	1 (7.7)	0
Cardiac arrest	0	0	1 (7.7)	0
Cardiac failure	0	0	1 (7.7)	0
Colitis	0	0	1 (7.7)	0
Delirium	0	0	0	1 (16.7)
Femur fracture	0	0	1 (7.7)	0
Gastrointestinal hemorrhage	0	0	1 (7.7)	0
Intestinal perforation	0	0	1 (7.7)	0
Pelvic pain	0	0	0	1 (16.7)
Pleural effusion	0	0	0	1 (16.7)
Seizure	0	0	0	1 (16.7)
Urinary tract infection	0	0	1 (7.7)	0

[a]Only AEs occurring during treatment or within 30 days of the last study medication were reported.

AE, adverse event; SAE, serious adverse event.

Supplementary Table S16. Overview of dose reductions (safety set, Part II)

n (%)	Encorafenib + binimetinib + ribociclib[a] *n*=38			Encorafenib + binimetinib + infigratinib[a] *n*=1			Encorafenib + binimetinib + capmatinib[a] *n*=13			Encorafenib + binimetinib + buparlisib[a] *n*=6		
	**Enco**	**Bini**	**Ribo**	**Enco**	**Bini**	**Infi**	**Enco**	**Bini**	**Cap**	**Enco**	**Bini**	**Bupa**
Without dose reduction	37 (97.4)	27 (71.1)	37 (97.4)	1 (100)	1 (100)	1 (100)	13 (100)	8 (61.5)	6 (46.2)	5 (83.3)	3 (50.0)	5 (83.3)
With at least one dose reduction	1 (2.6)	11 (28.9	1 (2.6)	0	0	0	0	5 (38.5)	7 (53.8)	1 (16.7)	3 (50.0)	1 (16.7)
With only one dose reduction	1 (2.6)	8 (21.1)	1 (2.6)	0	0	0	0	2 (15.4)	3 (23.1)	0	1 (16.7)	1 (16.7)
With more than one dose reduction	0	3 (7.9)	0	0	0	0	0	3 (23.1)	4 (30.8)	1 (16.7)	2 (33.3)	0

With at least one dose reduction by reason												
As per protocol	0	1 (2.6)	0	0	0	0	0	0	0	0	0	0
AE	0	7 (18.4)	1 (2.6)	0	0	0	0	4 (30.8)	6 (46.2)	0	2 (33.3)	1 (16.7)
Disease progression	0	1 (2.6)	0	0	0	0	0	1 (7.7)	1 (7.7)	0	0	0
Dosing error	0	2 (5.3)	0	0	0	0	0	2 (15.4)	2 (15.4)	0	1 (16.7)	0
Physician decision	0	0	0	0	0	0	0	1 (7.7)	0	0	0	0
Patient/guardian decision	0	2 (5.3)	0	0	0	0	0	0	0	0	0	0
Technical problems	0	1 (2.6)	0	0	0	0	0	0	0	0	1 (16.7)	0
Missing	1 (2.6)	0	0	0	0	0	0	0	0	1 (16.7)	0	0

[a]A patient with multiple dose reductions was only counted once in each dose reduction category. Each patient could have multiple reasons for dose reduction.

AE, adverse event; bini, binimetinib; bupa, buparlisib; cap, capmatinib; enco, encorafenib; infi, infigratinib; ribo, ribociclib.

Supplementary Table S17. Overview of dose interruptions (safety set; Part II)

n (%)	Encorafenib + binimetinib + ribociclib[a] *n*=38			Encorafenib + binimetinib + infigratinib[a] *n*=1			Encorafenib + binimetinib + capmatinib[a] *n*=13			Encorafenib + binimetinib + buparlisib[a] *n*=6		
	**Enco**	**Bini**	**Ribo**	**Enco**	**Bini**	**Infi**	**Enco**	**Bini**	**Cap**	**Enco**	**Bini**	**Bupa**
Without dose interruption	24 (63.2)	25 (65.8)	27 (71.1)	1 (100)	1 (100)	1 (100)	9 (69.2)	8 (61.5)	8 (61.5)	4 (66.7)	3 (50.0)	4 (66.7)
With at least one dose interruption	14 (36.8)	13 (34.2)	11 (28.9)	0	0	0	4 (30.8)	5 (38.5)	5 (38.5)	2 (33.3)	3 (50.0)	2 (33.3)
With only one dose interruption	10 (26.3)	10 (26.3)	6 (15.8)	0	0	0	1 (7.7)	1 (7.7)	2 (15.4)	1 (16.7)	2 (33.3)	2 (33.3)
With more than one dose interruption	4 (10.5)	3 (7.9)	5 (13.2)	0	0	0	3 (23.1)	4 (30.8)	3 (23.1)	1 (16.7)	1 (16.7)	0

With at least one dose interruption by reason												
AE	9 (23.7)	8 (21.1)	6 (15.8)	0	0	0	3 (23.1)	3 (23.1)	4 (30.8)	1 (16.7)	2 (33.3)	1 (16.7)
Dosing error	1 (2.6)	0	2 (5.3)	0	0	0	1 (7.7)	1 (7.7)	1 (7.7)	1 (16.7)	1 (16.7)	1 (16.7)
Physician decision	4 (10.5)	4 (10.5)	4 (10.5)	0	0	0	1 (7.7)	2 (15.4)	1 (7.7)	0	0	0
Patient/ guardian decision	1 (2.6)	1 (2.6)	1 (2.6)	0	0	0	1 (7.7)	1 (7.7)	1 (7.7)	0	0	0

[a]A patient with multiple dose interruptions was only counted once in each category. Each patient could have multiple reasons for dose interruption.

AE, adverse event; bini, binimetinib; bupa, buparlisib; cap, capmatinib; enco, encorafenib; infi, infigratinib; ribo, ribociclib.

Supplementary Table S18. DLTs observed during Cycle 1 (dose-determining set; Part II)[a,b,c,d]

**Primary system organ class preferred term, *n* (%)**	**Encorafenib + binimetinib + ribociclib *n*=34**	**Encorafenib + binimetinib + infigratinib *n*=0**	**Encorafenib + binimetinib + capmatinib *n*=12**	**Encorafenib + binimetinib + buparlisib *n*=0**
Any primary system organ class				
Grade 1	0		0	
Grade 2	0		0	
Grade 3	0		0	
Grade 4	1 (2.9)		0	
Investigations				
Grade 1	0		0	
Grade 2	0		0	
Grade 3	0		0	
Grade 4	1 (2.9)		0	
Amylase increase				

Grade 1	0		0	
Grade 2	0		0	
Grade 3	0		0	
Grade 4	1 (2.9)		0	
Lipase increase				
Grade 1	0		0	
Grade 2	0		0	
Grade 3	0		0	
Grade 4	1 (2.9)		0	

[a]The dose-determining set consisted of patients in the safety set from Part II who met the requirements for minimum safety evaluation and minimum exposure or experienced DLT during the first cycle of the assigned triple combination treatment.

[b]A patient with multiple DLTs within a primary system organ class was counted only once in the total row.

[c]A patient with multiple occurrences of DLTs under one treatment group was counted only once in the AE category for that treatment group.

[d]The on-treatment period was defined as the time from the first dose of study treatment through a minimum of 30 calendar days after the last administration of encorafenib or binimetinib study treatment.

AE, adverse event; DLT, dose limiting toxicity.
